# Betulinyl Sulfamates as Anticancer Agents and Radiosensitizers in Human Breast Cancer Cells

**DOI:** 10.3390/ijms161125953

**Published:** 2015-11-03

**Authors:** Matthias Bache, Christin Münch, Antje Güttler, Henri Wichmann, Katharina Theuerkorn, Daniel Emmerich, Reinhard Paschke, Dirk Vordermark

**Affiliations:** 1 Department of Radiotherapy, Martin Luther University Halle-Wittenberg, Ernst-Grube-Straße 40, D-06120 Halle, Germany; muench.christin@web.de (C.M.); antje.hahnel@uk-halle.de (A.G.); henri.wichmann@uk-halle.de (H.W.); katharina.theuerkorn@uk-halle.de (K.T.); dirk.vordermark@medizin.uni-halle.de (D.V.); 2 Biozentrum, Martin Luther Universität Halle-Wittenberg, Weinbergweg 22, D-06120 Halle, Germany; daniel.emmerich@student.uni-halle.de (D.E.); reinhard.paschke@biozentrum.uni-halle.de (R.P.)

**Keywords:** betulinic acid derivatives, carbonic anhydrase inhibitors, breast cancer, cytotoxicity, irradiation, normoxia, hypoxia

## Abstract

Betulinic acid (BA), a natural compound of birch bark, is cytotoxic for many tumors. Recently, a betulinyl sulfamate was described that inhibits carbonic anhydrases (CA), such as CAIX, an attractive target for tumor-selective therapy strategies in hypoxic cancer cells. Data on combined CAIX inhibition with radiotherapy are rare. In the human breast cancer cell lines MDA-MB231 and MCF7, the effects of BA and betulinyl sulfamates on cellular and radiobiological behavior under normoxia and hypoxia were evaluated. The two most effective betulinyl sulfamates CAI 1 and CAI 3 demonstrated a 1.8–2.8-fold higher cytotoxicity than BA under normoxia in breast cancer cells, with IC_50_ values between 11.1 and 18.1 µM. BA exhibits its strongest cytotoxicity with IC_50_ values of 8.2 and 16.4 µM under hypoxia. All three substances show a dose-dependent increase in apoptosis, inhibition of migration, and inhibition of hypoxia-induced gene expression. In combination with irradiation, betulinyl sulfamates act as radiosensitizers, with DMF10 values of 1.47 (CAI 1) and 1.75 (CAI 3) under hypoxia in MDA-MB231 cells. BA showed additive effects in combination with irradiation. Taken together; our results suggest that BA and betulinyl sulfamates seem to be attractive substances to combine with radiotherapy; particularly for hypoxic breast cancer.

## 1. Introduction

Surgery, radiotherapy and systemic chemotherapy are the main forms of therapy for breast cancer. Further treatments, such as the therapeutic antibody trastuzumab and hormone therapy, complement these therapies. Using multimodal therapy concepts, the mortality rate of breast cancer patients is reduced. However, breast cancer remains the most prevalent cause of cancer-specific death in women. Additional tumor-selective treatment strategies are necessary to improve the treatment success of breast cancer patients.

Betulinic acid (BA) is a natural substance of birch bark with broad anti-tumor properties, such as high cytotoxicity, induction of apoptosis, and inhibition of migration and antiangiogenic effects. BA is cytotoxic in many types of tumors *in vitro* [1]. Moreover, Zuco *et al.* found an increased cytotoxicity in cancer cells compared to normal cells such as fibroblasts and lymphocytes [2]. Furthermore, *in vivo* studies showed an inhibition of tumor growth without systemic toxicity [3]. Additionally, BA is also an effective anticancer agent in breast cancer cells [4]. Recently, a therapeutic benefit for breast cancer was demonstrated *in vivo* [5,6,7]. Selective cytotoxic effects of BA suggest a potential benefit from combination with chemotherapy or radiotherapy [8]. Different studies have shown synergistic effects of BA with different chemotherapeutics in various tumor cell lines [9]. Initial studies with selected melanoma [10] and head-and-neck [11] tumor cell lines illustrated additive effects of BA and irradiation [12]. Our own results demonstrated that BA increased the cytotoxic and radiosensitizing effects in human glioma cells under hypoxia [8]. This increase was associated with a decrease in the hypoxia-induced protein levels of HIF-1α, the most important oxygen sensor in mammalian cells. Other studies confirmed the inhibitory effects of BA on the expression of hypoxia-induced genes [13,14,15].

The disadvantage of BA for use in tumor therapy is its low solubility. To address this issue, different formulations have been used as an approach to facilitate the use of BA in tumor therapy. Recently, a randomized phase II study of betulin-based Oleogel-S10 demonstrated no effects in the treatment of patients with actinic keratosis [16]. Up to now, no successful clinical trial has been conducted that supports the use of BA for the treatment of human cancer patients. Modifications of BA open the possibility to develop substances with optimized properties for targeted tumor therapy. Recently, studies confirm that BA derivatives, such as NVX-207 or B10, have an increased cytotoxic activity under normoxia [17,18,19]. However, compared to BA, our own results identified lower effects of NVX-207 or B10 under hypoxia [12]. Winum *et al.* described betulin 3,28-disulfamate, a BA derivative that acts as a carbonic anhydrase inhibitor (CAI) [20]. In addition to the ability of CA inhibition, sulfamates exhibit further therapeutic potential through the inhibition of additional targets, such as aminoacyl-tRNA synthetases or steroid sulfatases, of breast cancer patients [21].

CA catalyzes the hydrogenation of CO_2_ to HCO_3_^−^ and H^+^ and regulates the intracellular and extracellular pH of cells. CA seems to be important for the development, malignant potential, and metastasis of solid tumors. The high metabolic activity of tumors leads to hypoxia and acidosis, especially in poorly vascularized tumor regions. Nearly 50% of locally advanced breast cancers exhibit hypoxic and/or anoxic regions [22]. Carbonic anhydrase IX (CAIX), a member of the CA family, is a transmembrane protein and one of the most stable HIF-1α-regulated proteins. In various tumor types, such as lung, cervical, head-and-neck or breast cancer, high CAIX expression levels are closely associated with a poor prognosis [23]. CAIX inhibition is therefore an attractive target for tumor-selective treatment strategies [24]. However, data combining CA inhibition and radiotherapy are limited so far.

In the present study, we analyzed the cellular- and radio-biological effects of BA, betulin 3,28-disulfamate, and three newly developed betulinyl sulfamates under normoxia and hypoxia in human breast cancer cells.

## 2. Results and Discussion

### 2.1. Results

#### 2.1.1. Effects of BA and Betulinyl Sulfamates on the Cytotoxicity, Clonogenic Survival, Apoptosis and Migration in Breast Cancer Cell Lines

**Figure 1:**
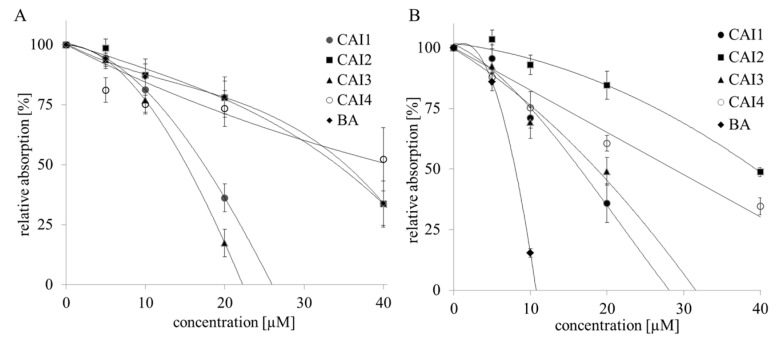
Cytotoxicity of BA and betulinyl sulfamates for MDA-MB231 cells, determined using the Sulforhodamine-B (SRB)-assay. Cells were treated using increasing doses of BA, CAI 1, CAI 2, CAI 3 or CAI 4 under normoxic (**A**) or hypoxic (**B**) conditions. The data represent the mean values (±SD) of three independent experiments.

**Figure 2:**
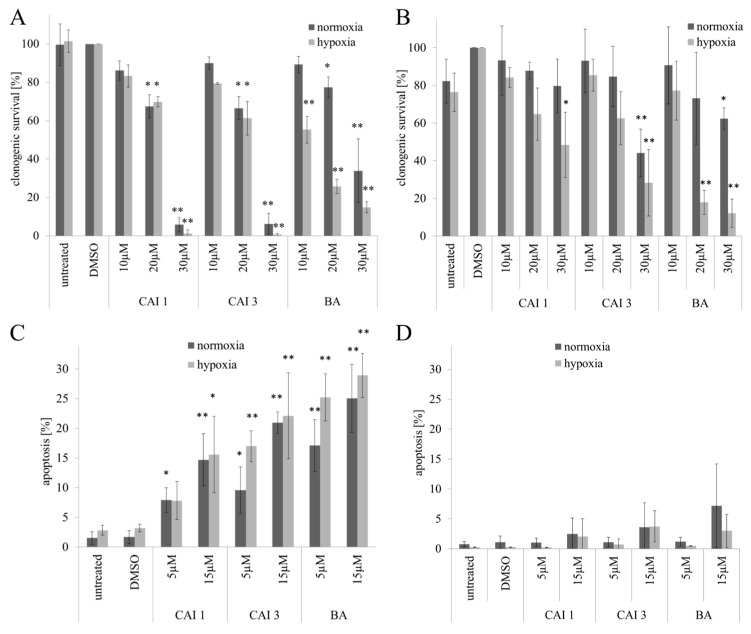
Effects of BA, CAI 1 and CAI 3 incubation on clonogenic survival (**A**,**B**) and apoptosis (DAPI staining) (**C**,**D**) in MDA-MB231 **(A,C**) und MCF7 (**B,D**) cells. The data represent the mean values (±SD) of three to five independent experiments. Significant differences to dimethyl sulfoxide (DMSO) treatment were labeled (* *p* < 0.05, ** *p* < 0.001).

The IC_50_ values of the betulinyl sulfamates differed, ranging between 13.6 and 44.5 µM in MDA-MB231 or 11.1 and 31.3 µM in MCF7 cells under normoxia as determined with the Sulforhodamine-B (SRB) assay. CAI 1 and CAI 3, the most effective betulinyl sulfamates, demonstrated a 1.8–2.8-fold higher cytotoxicity than did BA under normoxia, which showed a moderate cytotoxicity, with IC_50_ values of 32.1 and 31.5 µM in MDA-MB231 and MCF7 cells, respectively. Compared to normoxia, a similar or lower cytotoxicity of betulinyl sulfamates (CAI 1–4) in MDA-MB231 or MCF7 cells was observed under hypoxia (Table 1). BA exhibited the highest cytotoxicity, with IC_50_ values of 8.2 and 16.4 µM in both of the breast cancer cell lines, respectively, compared to the betulinyl sulfamates under hypoxic conditions (Table 1, Figure 1). Similar effects were observed using clonogenic survival assays (Figure 2).

**Table 1:** IC_50_ values of BA and betulinyl sulfamates for human breast cancer cell lines.

Drug	MDA-MB231 Cells		MCF7 Cells	
	Normoxia	Hypoxia	Normoxia	Hypoxia
CAI 1	18.1 ± 2.9 μM	16.7 ± 2.6 μM	12.6 ± 0.4 μM	28.7 ± 2.6 μM
CAI 2	33.3 ± 3.9 μM	37.7 ± 2.0 μM	31.3 ± 1.8 μM	>40 μM
CAI 3	13.6 ± 1.6 μM	19.5 ± 1.5 μM	11.1 ± 0.9 μM	30.9 ± 1.9 μM
CAI 4	>40 μM	26.1 ± 2.9 μM	24.3 ± 1.4 μM	>40 μM
BA	32.1 ± 4.3 μM	8.2 ± 0.2 μM	31.5 ± 1.7 μM	16.4 ± 3.7 μM

IC_50_—half maximal inhibitory concentration.

**Figure 3:**
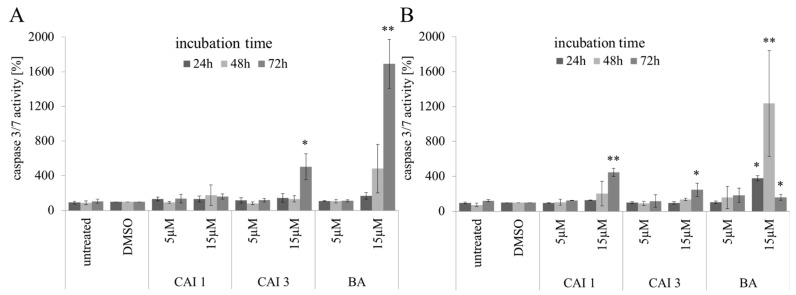
Effects of BA, CAI 1 and CAI 3 on caspase 3/7 activation in MDA-MB231 cells under normoxic (**A**) or hypoxic (**B**) conditions. The data represent the mean values (±SD) of three independent experiments. Significant differences to DMSO treatment were labeled (* *p* < 0.05, ** *p* < 0.001).

To investigate rates of apoptosis, nuclear staining with DAPI and caspase 3/7 activation was used. Compared to betulinyl sulfamates, BA possessed the strongest effects on the induction of apoptosis in breast cancer cells when evaluating apoptosis with DAPI staining. BA, CAI 1, and CAI 3 exhibited a similar dose-dependent increase in apoptosis under normoxia and hypoxia. However, BA or betulinyl sulfamate treatment resulted in a distinct induction of apoptosis only in MDA-MB231 cells after an incubation time of 48 h (Figure 2). In caspase 3 defective MCF7 cells the incubation with 15 µM BA or betulinyl sulfamates resulted in a weak induction of apoptosis of 1.4% to 6.2%, as verified by DAPI-staining. In MDA-MB231 cells, however, a significant induction in apoptosis was detected using 5 µM of the substances between 4.6% and 22.0%. At a concentration of 15 µM CAI 1, CAI 3 and BA, an increased rate of apoptosis between 12.4% and 25.7% was detected (Figure 2). BA also demonstrated the strongest caspase 3/7 activation (17-fold compared to that of the dimethyl sulfoxide [DMSO] control), with a maximum after incubation for 72 h under normoxia (Figure 3). However, the caspase 3/7 activation (up to 5-fold compared to that of the DMSO control) of both of the betulinyl sulfamates was significantly lower compared to that of BA without obvious differences between normoxia and hypoxia. Under hypoxia, BA caused maximal caspase 3/7 activation (12-fold compared to that of the DMSO control) after 48 h incubation (Figure 3). After an incubation time of 72 h, the BA-induced caspase 3/7 activation decreased (1.6-fold compared to that of the DMSO control).

**Figure 4:**
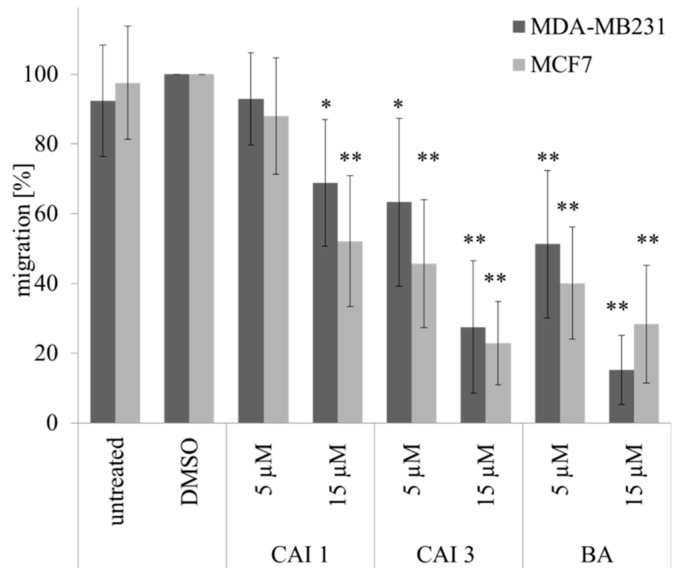
Effects of BA, CAI 1 and CAI 3 incubation on migration determined by scratch assay in MDA-MB231 und MCF7 cells. The data represent the mean values (±SD) of three independent experiments. Significant differences to DMSO treatment were labeled (* *p* < 0.05, ** *p* < 0.001).

The effects of BA, CAI 1, and CAI 3 on the cell migration of breast cancer cell lines were determined using a scratch assay. In both of the breast cancer cell lines, BA and CAI 3 showed stronger effects on the inhibition of migration than did CAI 1 (Figure 4). Both of the substances exhibited a significant effect on migration at a concentration of 5 µM. However, CAI 1 did not inhibit migration at a concentration of 5 µM (Figure 4). At 15 µM BA and CAI 3, the migration rate strongly decreased to 15.2% and 27.5% in MDA-MB231 cells and to 22.8% and 28.4% in MCF7 cells. At a concentration of 15 µM, CAI 1 moderately decreased the rate of migration to 68.8% and 52.1% in MDA-MB231 and MCF7 cells, respectively.

#### 2.1.2. Effects of BA and Betulinyl Sulfamates on the Protein Expression Using Western Blotting and Radiosensitivity in Breast Cancer Cells

**Figure 5:**
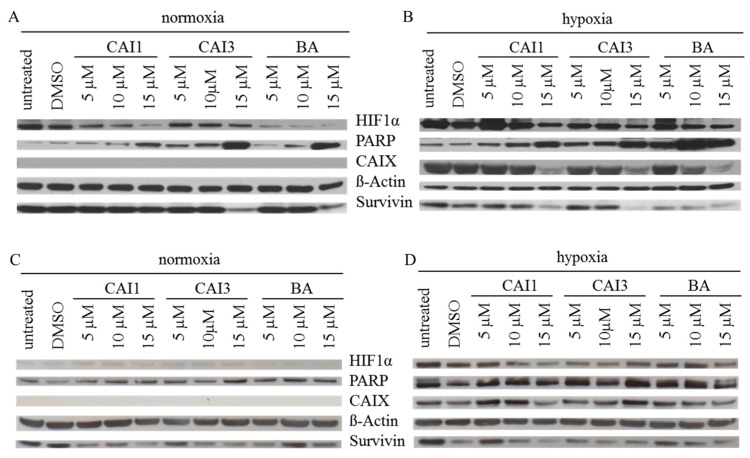
Effects of BA, CAI 1 and CAI 3 incubation on protein expression of HIF-1α, PARP, CAIX and survivin in MDA-MB231 (**A**,**B**) and MCF-7 (**C**,**D**) cells under normoxia (**A**,**C**) and hypoxia (**B**,**D**) using Western blotting. BA, CAI 1 and CAI 3 treatment increased PARP cleavage and decreased expression level of HIF-1α, CAIX and survivin in MDA-MB231 cells. However, effects in MCF7 cells were less obvious than in MDA-MB231 cells. β-Actin served as an internal loading control. For Western blot analysis, one representative result of three or four independent experiments is shown.

**Figure 6:**
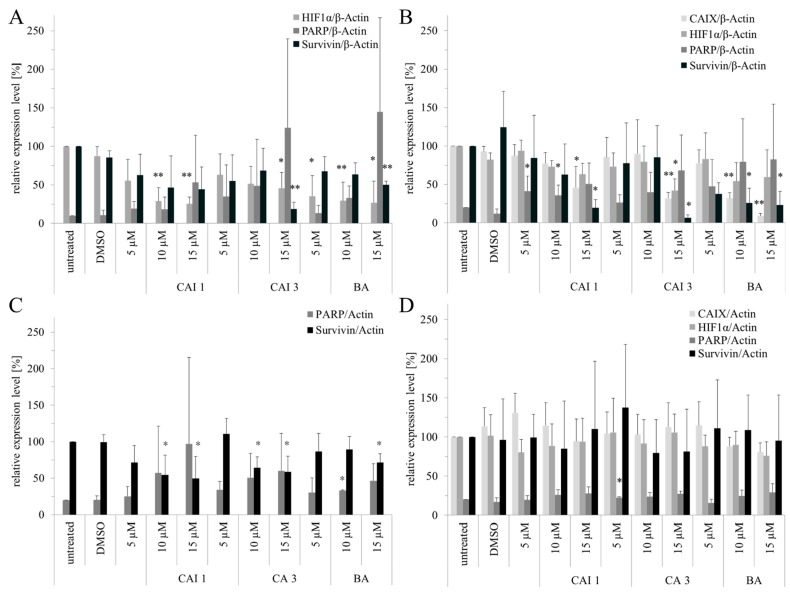
Effects of BA, CAI 1 and CAI 3 incubation on densitometric evaluated HIF-1α, PARP, CAIX and survivin protein levels in MDA-MB231 (**A**,**B**) and MCF-7 (**C**,**D**) cells under normoxia (**A**,**C**) and hypoxia (**B**,**D**) conditions. Western blots were analyzed with Aida Analysis 2D Software. β-Actin served as an internal loading control. Significant differences to DMSO treatment were labeled (* *p* < 0.05, ** *p* < 0.001).

In both breast cancer cell lines we examined the effects of BA, CAI 1, and CAI 3 on Poly ADP-ribose polymerase (PARP) cleavage and protein level of survivin and the hypoxia-induced genes HIF-1α and CAIX under normoxia and hypoxia (Figure 5). Densitometric evaluation and *p*-values of the protein expression data are presented in Figure 6. Under normoxia no CAIX and low HIF-1α protein levels were detected. As expected, hypoxia induced the expression of HIF-1α and CAIX. In addition, hypoxia led to a weak increase in cleaved PARP and a decrease in the protein level of survivin. Incubation with each of the substances resulted in a dose-dependent increase in PARP cleavage and a decrease in the protein level of survivin under normoxia and hypoxia. In addition, both of the hypoxia-induced genes HIF-1α and CAIX decreased in a dose-dependent manner in response to BA, CAI 1, and CAI 3 incubation in MDA-MB231 cells. Compared to MDA-MB231 cells these effects were less obvious in MCF7 cells. In addition, the effects of BA, CAI 1, and CAI 3 on protein expression were only partially significant (Figure 6).

**Figure 7:**
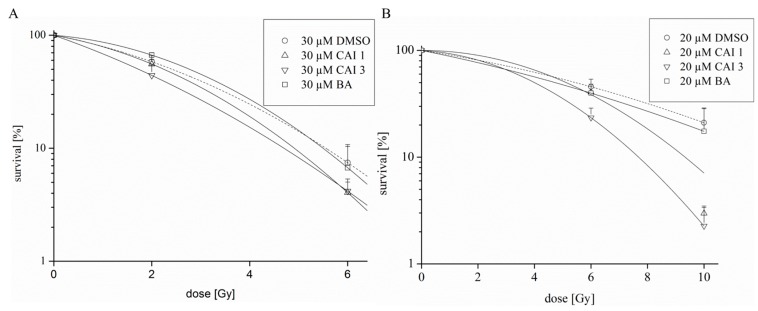
Effects of BA, CAI 1, and CAI 3 on the radiosensitivity of human breast cancer cells. MDA-MB231 cells were treated with BA, CAI 1, and CAI 3 and irradiated with a dose of 2, 6 or 10 Gy under normoxia (**A**) or hypoxia (**B**). The data represent the mean values (±SD) of three independent experiments.

We determined the radiosensitivity of BA, CAI 1 or CAI 3 after irradiation with 2, 4, 6 or 10 Gy in MDA-MB231 cells under normoxia and hypoxia. Based on the decreased clonogenic survival, 30 µM (normoxia) or 20 µM (hypoxia) of the substances was used. For BA, additive effects with irradiation, independent of the oxygen level, were obtained. Both of the betulinyl sulfamates had slightly increased effects on the radiosensitivity of MDA-MB231 cells under normoxia (Figure 7). The DMF10 values of CAI 1 and CAI 3 were 1.15 and 1.20, respectively. With DMF10 values of 1.47 for CAI 1 (*p* = 0.077) and 1.75 for CAI 3 (*p* = 0.028), the effects on radiosensitivity were stronger for both of the betulinyl sulfamates under hypoxic conditions (Table 2).

**Table 2:** DMF10 values of BA, CAI 1, or CAI 3 for the human breast cancer cell line MDA-MB231.

MDA-MB231 Cells					
Normoxia			Hypoxia		
Drug	DMF10	*p*-Value	Drug	DMF10	*p*-Value
30 µM CAI 1	1.15	0.078	20 µM CAI 1	1.47	0.077
30 µM CAI 3	1.20	0.035 *	20 µM CAI 3	1.75	0.028 *
30 µM BA	1.01	0.922	20 µM BA	1.06	0.920

DMF10—dose-modifying factor at the 10% survival level, Significant differences were labeled (* *p* < 0.05).

### 2.2. Discussion

Betulinic acid (BA), a natural compound of birch bark, demonstrates antitumor effects in different tumor entities *in vitro*. Due to its poor solubility, BA is not currently used for the clinical treatment of tumors. Specific modifications could be an attractive approach for the use of BA in targeted tumor therapy [12]. Winum *et al.* described betulin 3,28-disulfamate, a BA derivative that acts as an effective CAI [20]. The inhibition of CAIX is a prominent strategy for the selective therapy of hypoxic, radioresistant tumors. In the present study, we analyzed the cellular- and radio-biological effects of BA, betulin 3,28-disulfamate and the new synthesized betulinyl sulfamates on human breast cancer cell lines under normoxia and hypoxia.

Compared to BA, the betulinyl sulfamates CAI 1 and CAI 3 showed a 1.8–2.8-fold higher cytotoxicity in the breast cancer cell lines MDA-MB231 and MCF7 under normoxia (Table 1). Recently, a study evaluated 28-acetyl-3-sulfamoyloxybetulin (CAI 2) and other pentacyclic triterpenoide sulfamates as suitable cytotoxic components in different tumor cell lines, as shown by the SRB assay [25]. In contrast, S4, a sulfamate specifically inhibiting CAIX/CAXII, demonstrated no effect on the tumor growth of MDA-MB231 breast cancer cells *in vitro* or *in vivo* [26]. The results of the SRB assay suggest that betulinyl sulfamates have a similar or lower cytotoxicity under hypoxic conditions (Figure 1). Strongly delayed cell growth of hypoxic control cells seems to be responsible for the apparent resistance of cancer cells [12]. Our analysis with SRB assays showed stronger cytotoxicity under hypoxia, particularly for BA (Table 1, Figure 1). This result is in agreement with our data of clonogenic survival (Figure 2). Recently, we detected a stronger cytotoxicity of BA for glioma cell lines under hypoxia [8,12]. When evaluating additional anti-cancer properties of CAI 1, CAI 3 or BA, the induction of apoptosis and the inhibition of migration in breast cancer cells was observed (Figure 2, Figure 3 and Figure 4). BA and both of the CAIs induced caspase 3/7 activation and apoptosis in MDA-MB231 cells. In addition, our analysis showed that CAI 1, CAI 3, and BA induced PARP cleavage and decreased the protein level of survivin (Figure 5 and Figure 6). Previous studies also detected BA- or CAI-induced apoptosis in breast cancer cell lines [1,6,27,28,29,30]. However, CAI 1 and CAI 3 incubation only induced a low caspase 3/7 activation (Figure 3). Moreover, apoptosis induction was detected in caspase 3 defective MCF7 cells (Figure 2). Recently, caspase-independent forms of BA-induced cell death were discussed [31]. Consistent with this result, Lou *et al.* suggested caspase-independent apoptosis induction by CAIX shRNA [29]. In addition, both BA and CAI incubation resulted in the autophagy of human breast cancer cells [31,32]. In different studies, a BA-induced inhibition of migration was detected [8,33,34]. Comparable to BA, the CAI 1 and CAI 3 treatments resulted in reduced rates of migration of both breast cancer cells lines (Figure 4). The inhibition of carbonic anhydrases with the non-specific inhibitor acetazolamide or CAIX siRNAs results in a decrease of migration and invasion in breast cancer cells [35]. Consistent with this result, CAI S4 reduced migration and metastasis in breast cancer cells *in vitro* and *in vivo* [26]. Furthermore, CA9 transfection or CA9 siRNAs modulate the invasive and metastatic ability of cervical carcinoma cells [36]. Analyzing hypoxia-induced genes, we found that BA, CAI 1 and CAI 3 reduced the HIF-1α and CAIX levels in breast cancer cells (Figure 6). The mRNA level of CA9, but not HIF-1α, was also reduced by an incubation of BA and betulinyl sulfamates (data not shown). In agreement with this result, other CA inhibitors reduced the CA9 mRNA and protein levels in human glioma cell lines [37,38]. Considering the reduced CAIX levels, the CAI-promoted internalization and degradation of CAIX has been discussed [39]. In further studies, BA treatment resulted in HIF-1α inhibition or anti-angiogenic activity [13,14,15]. Shin *et al.* reported a STAT3-induced HIF-1α inhibition because BA treatment inhibited hypoxia-mediated STAT3 phosphorylation. Nevertheless, the mechanisms of the BA-induced inhibition of HIF-1α remain unclear [14]. In agreement, we previously demonstrated a decreased BA-induced HIF-1α protein level in glioma cells [8]. These data suggest that BA and betulinyl sulfamates exhibit a strong specificity in inhibiting the hypoxia-induced CAIX pathway. Nevertheless, the permeation of BA and BA derivatives into the hypoxic tumor region is a major issue for their therapeutic use, which should be a subject of future studies.

Comparing both breast cancer cell lines, BA and betulinyl sulfamates induced only weak effects on HIF-1α and CAIX expression and PARP cleavage in MCF7 cells (Figure 5 and Figure 6). This is in agreement with somewhat weaker effects of BA and BA sulfamates on cytotoxicity and apoptosis in MCF7 cells (Figure 2). A recent study also detected a reduced inhibition of spheroid formation in CAIX-shRNA transfected MCF7 cells [40]. Restoration of caspase 3 in this breast cancer cell line increased the rate of apoptosis in response to doxorubicin and other chemotherapeutics [41,42]. Maybe caspase-3 deficiency has an impact on the slightly decreased effects of BA and betulinyl sulfamates in MCF7 cells.

However, CAI 1 and CAI 3 (but not BA) affect the radiosensitivity of MDA-MB231 cells, particularly under hypoxia (Figure 7). It is likely that betulinyl sulfamates exhibit a stronger CAIX inhibition, with additional effects as CAI compared to BA. Consistent with the radiosensitization by betulinyl sulfamates, further studies from our group detected the radiosensitization of CA9-siRNA treated MDA-MB231 cells (Theuerkorn *et al.* in preparation). Moreover, the stable silencing of CA9 or CA12 by shRNA resulted in an induction of cell death and radiosensitization of colon carcinoma cells [43]. Similarly, a sulfamide of the hypoxic radiosensitizer nitroimidazole enhanced the effects of irradiation of colorectal cancer *in vivo* in a CAIX-dependent manner [44].

## 3. Experimental Section

### 3.1. Cell Lines, Treatments and Irradiation

**Figure 8:**
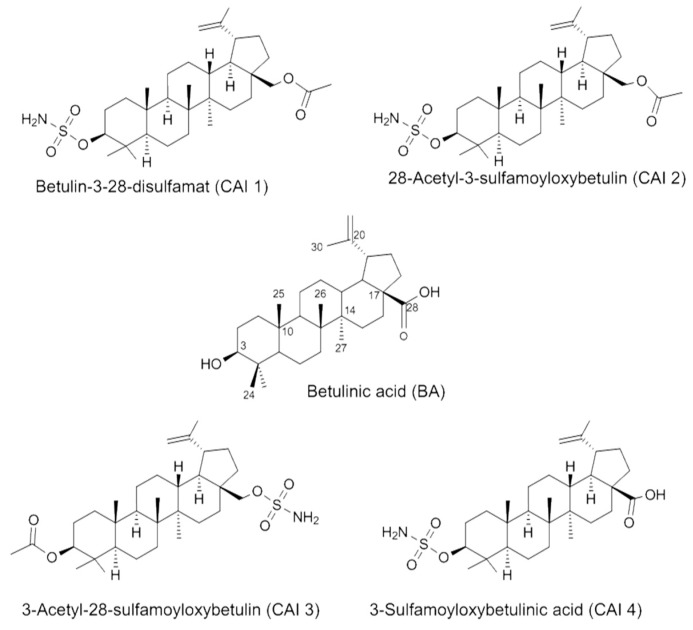
Structures of betulinic acid (BA), CAI 1–4.

The human breast cancer cell lines MDA-MB231 and MCF7 (kindly provided by Jürgen Dittmer, Department of Gynecology, University Halle-Wittenberg, Halle, Germany) were grown in RPMI 1640 medium (Lonza, Walkersville, MD, USA) containing 10% fetal bovine serum (PAA Laboratories, Cölbe, Germany), 1% sodium pyruvate (Invitrogen, Karlsruhe, Germany), 185 U/mL penicillin (Invitrogen) and 185 μg/mL streptomycin (Invitrogen) at 37 °C in a humidified atmosphere containing 3% CO_2_. Hypoxia (<0.1% O_2_) was achieved using a gas generator system (Anaerocult^®^ P Merck, Darmstadt, Germany). The chemical structures of BA, betulin 3,28-disulfamate (CAI 1) and the newly developed betulinyl sulfamates (CAI 2–4) are shown in Figure 8. Computer simulations have shown that the solvent accessibility is increased by the introduction of the polar sulfamate group. This was demonstrated by HPLC measurements. Compounds CAI 1–CAI 4 were synthesized according to literature methods and new procedures that will be published separately (Paschke *et al.*, in preparation). BA was a gift from BioSolutions Halle, Germany. All of the compounds were dissolved in dimethyl sulfoxide (DMSO) to achieve a 20 mM stock solution. The cells were treated with BA or CAI 1–4 for 24 h at 37 °C under normoxic or hypoxic conditions and were subsequently irradiated. Irradiation was performed using 6-MV photons, adequate bolus material, and a linear accelerator (SIEMENS ONCOR, Erlangen, Germany) at a dose rate of 2 Gy/min as previously described [12].

### 3.2. SRB Assay

The cytotoxic activities were evaluated using the Sulforhodamine-B (SRB) assay as previously described [12]. Briefly, exponentially growing cells were seeded into 96-well plates for 24 h, after which the cells were treated using a dilution series of BA and CAI 1–4 for 72 h under normoxia or hypoxia. After treatment, the cells were fixed using 10% TCA at 4 °C for 1 h, washed with ice-cold water and dyed using 0.4% SRB solution (Sigma, Steinheim, Germany) for 10 min. After staining, the plates were washed with 1% acetic acid and air-dried overnight. A 20-mM Tris base solution was added, and the absorbance was measured at 540 nm using a 96-well plate reader (TECAN GENios, Männedorf, Switzerland). The IC_50_ values indicate the half maximal inhibitory concentration of the compounds. The data were obtained from three independent experiments.

### 3.3. Caspase 3/7 Assay

After incubation for 24 h, 48 h or 72 h with BA, CAI 1 or CAI 3, the activation of caspase 3 and 7 was measured with the Caspase-Glo^®^3/7 assay (Promega, Madison, WI, USA) as previously described [45]. Briefly, the PBS-washed cells were incubated for 30 min in a buffer-substrate mix, and the luminescent signal was measured with a 96-well plate reader (TECAN, Grödig, Austria). The results represent at least three independent experiments.

### 3.4. DAPI Staining

A morphological analysis of apoptosis was performed using DAPI (4,6-diamidino-2-phenylindole dihydrochloride) staining as previously described [46]. Briefly, we analyzed the adherent cells and supernatant 48 h after incubation with BA, CAI 1, or CAI 3. The cells were fixed with 80% ethanol (Merck, Darmstadt, Germany) and centrifuged on microscope slides at 1000× *g* for 5 min. After staining with DAPI solution (Serva, Heidelberg, Germany), the cells were covered with Prolong^®^ Gold Antifade (Invitrogen). Apoptotic cells were determined according to nuclear condensation and fragmentation with a fluorescent microscope at 200× magnification (BZ-8000, microscope, Keyence, Japan). The data represent the results of at least three independent experiments.

### 3.5. Scratch Assay

A scratch assay was used to determine the migration of cells as previously described [12]. Briefly, the cells were grown in 24-well cell culture plates to 100% confluence. A uniform, cell-free area was created by scratching the confluent monolayer. To determine the migration of breast cancer cells, the wound closure was observed 16 h after incubation with BA, CAI 1 or CAI 3. To evaluate the migration rate, the initial and the final wound areas were measured using Adobe Photoshop CS6. The data represent the results of at least three independent experiments.

### 3.6. Western Blotting

The protein expression levels of hypoxia-induced and apoptosis-associated genes were evaluated using Western blotting as previously described [47]. Briefly, after incubation for 48 h with BA, CAI 1 or CAI 3, the cells were washed and lysed with 50 µL of lysis-buffer (50 mM Tris-HCl, 200 mM NaCl, 1 mM EDTA, 1 mM EGTA, 1% TritonX-100, 0.25% deoxycholate, protease and phosphatase inhibitors) followed by ultrasonic homogenization. The protein concentrations were determined using the Bradford assay (Bio-Rad, Munich, Germany). Approximately, 60 µg of total protein was separated by electrophoresis in 4%–12% Gels (Invitrogen) and transferred to a Polyvinylidene difluoride (PVDF) membrane (Millipore, Schwalbach, Germany) by tank-blotting (Bio-Rad, München, Germany). After blocking, the PVDF membrane was incubated with the primary antibody with rabbit survivin antibody (1:1000 dilution, clone AF886, R&D Systems, Wiesbaden, Germany), rabbit cleaved PARP (1:2000, Cell Signaling, Danvers, MA, USA), mouse CAIX antibody (1:3000, BioScience, Bratislava, Slovakia), mouse HIF-1α antibody (1:2000, BD Biosciences, Heidelberg, Germany) and mouse anti-β-actin (1:5000, Sigma, Deisenhofen, Germany) at 4 °C overnight. The membranes were incubated with a horseradish peroxidase-labeled goat anti-rabbit IgG or anti-mouse IgM (1:2000, DAKO, Glostrup, Denmark) for 1 h at room temperature. For protein detection, the membranes were incubated with ECL Prime Western Blotting Detection reagents (GE Healthcare, Little Chalfont, UK) and exposed to CL-XPosure film (Thermo Fisher Scientific, Schwerte, Germany). The Western blot analysis is shown as one representative result from three independent experiments.

### 3.7. Clonogenic Survival Assay and Radiosensitivity

The clonogenic survival was evaluated as previously described [12]. Briefly, 24 h after treatment with BA, CAI 1 or CAI 3 (1 h after irradiation), the cell numbers were determined. Based on the optimal plating efficacy, 400–12,000 cells were seeded in 25-cm^2^ flasks between 10 and 14 days after irradiation. The cells were fixed using paraformaldehyde (Sigma, Deisenhofen, Germany) for 20 min and stained with 10% Giemsa solution (Sigma, Deisenhofen, Germany) for 20 min. Only colonies with >50 cells were scored to determine the surviving fraction (SF). The dose-modifying factor at the 10% survival level (DMF10) was determined to analyses the effects of BA, CAI 1 or CAI 3 on the radiosensitivity (DMF10 was defined as the ratio of radiation dose at the 10% survival level of untreated cells and radiation dose at the 10% survival level of treated cells) [47]. The data represent at least three independent experiments.

### 3.8. Statistical Analyses

The normal distribution of the experimental results was verified with Shapiro–Wilk-test. Depending on the normal distribution, the results were analyzed using Student’s *t*-tests or Mann–Whitney-*U*-tests. A *p*-value of 0.05 was considered statistically significant.

## 4. Conclusions

BA and betulinyl sulfamates affect the cytotoxicity, migration, apoptosis and/or radiosensitivity of human breast cancer cells. In particular, BA, CAI 1, or CAI 3 resulted in a higher cytotoxicity or radiosensitivity under hypoxia. Based on the radiation resistance of hypoxic breast cancer cells BA and betulinyl sulfamates are promising drugs in combination with radiotherapy for the treatment of breast cancer. Further studies must demonstrate the potential contribution of betulinyl sulfamates for the radiation treatment of hypoxic tumors.
